# Steady-State Linear and Non-linear Optical Spectroscopy of Organic Chromophores and Bio-macromolecules

**DOI:** 10.3389/fchem.2018.00086

**Published:** 2018-04-03

**Authors:** Marco Marazzi, Hugo Gattuso, Antonio Monari, Xavier Assfeld

**Affiliations:** ^1^Laboratoire de Physique et Chimie Théoriques, Université de Lorraine–Nancy, UMR 7019, Vandoeuvre-lés-Nancy, France; ^2^Laboratoire de Physique et Chimie Théoriques, Centre National de la Recherche Scientifique, UMR 7019, Vandoeuvre-lès-Nancy, France; ^3^Departamento de Química, Centro de Investigacíon en Síntesis Química (CISQ), Universidad de La Rioja, Logroño, Spain

**Keywords:** nucleic acids, lipid membrane, (poly)peptide, circular dichroism, (two-photon) absorption, fluorescence, hybrid quantum mechanics/molecular mechanics, solvent and dynamics effects

## Abstract

Bio-macromolecules as DNA, lipid membranes and (poly)peptides are essential compounds at the core of biological systems. The development of techniques and methodologies for their characterization is therefore necessary and of utmost interest, even though difficulties can be experienced due to their intrinsic complex nature. Among these methods, spectroscopies, relying on optical properties are especially important to determine their macromolecular structures and behaviors, as well as the possible interactions and reactivity with external dyes—often drugs or pollutants—that can (photo)sensitize the bio-macromolecule leading to eventual chemical modifications, thus damages. In this review, we will focus on the theoretical simulation of electronic spectroscopies of bio-macromolecules, considering their secondary structure and including their interaction with different kind of (photo)sensitizers. Namely, absorption, emission and electronic circular dichroism (CD) spectra are calculated and compared with the available experimental data. Non-linear properties will be also taken into account by two-photon absorption, a highly promising technique (i) to enhance absorption in the red and infra-red windows and (ii) to enhance spatial resolution. Methodologically, the implications of using implicit and explicit solvent, coupled to quantum and thermal samplings of the phase space, will be addressed. Especially, hybrid quantum mechanics/molecular mechanics (QM/MM) methods are explored for a comparison with solely QM methods, in order to address the necessity to consider an accurate description of environmental effects on spectroscopic properties of biological systems.

## Introduction

The frontier between computational biochemistry and computational chemistry is now becoming blurred thanks to the development of novel more efficient modeling methods able to tackle very large systems also thanks to new more powerful hardware architectures. For a long time, biological systems, due to the enormous number of particles (atoms) to deal with, were studied only by means of statistical simulations based on classical force fields. Hence electronic phenomena were explicitly excluded from such studies. Contrarily, chemical systems were treated by means of quantum chemistry tools since electronic motion was involved. However, most calculations were done placing the system in isolated (infinitely diluted gas phase) conditions. Nowadays, taking into account surroundings effects, like solvent effects, on chemical reactivity is routinely achieved either by implicit methods (Ruiz-Lopez et al., 1993; Tomasi et al., 2005) or explicit one. Conversely, studying electronic phenomena in biological macromolecules is also widely spread. These enlargements of both field, make them quite similar from the methodological point of view, since one needs to combine high accuracy quantum chemical calculations with statistical thermodynamics simulations in order to get meaningful information on electronic phenomena (chemical reactions, photochemical reactions, or electronic spectroscopy) in large systems. The appearance of hybrid quantum mechanics/molecular mechanics methods (QM/MM) in the 90's is at the origin of this merging between chemistry and biology, as recognized by the 2013 Nobel Prize (The Nobel Prize in Chemistry, 2013)^1^. It opens the way toward a brand new unexplored field: tackling electronic excited states in complex systems.

In this review we gather several applications carried out mainly by our group during the past 5 years, or so, that deal with different electronic spectroscopies, namely linear absorption, non-linear spectroscopy [especially two-photon absorption (TPA)], and circular dichroism on chemical or biochemical systems. All the chosen applications have a very tight link to critical biological problems.

This review is organized as follows. In section Linear Absorption, the importance of the surroundings and of the dynamical effects on electronic absorption spectra will be presented for various DNA photosensitizers. This is particularly important since DNA, although quite stable (almost no absorption) with respect to visible light irradiation, can be damaged by energy/charge transfer from small neighboring molecules (photosensitizers) that absorb the visible light. Section non-linear Spectroscopy collected mainly our studies on TPA. This method, arising from material science, starts now to be widely employed in photodynamic therapy since it allows (i) a deeper penetration of the beam, thanks to the use of red or infrared wavelengths, and (ii) a more precise localization of the action, since the density of photons needed for a twin absorption is high enough only in the focal region of the laser beam. The various tenets of the simulations will be discussed and their validity highlighted by numerical results. Finally, in section Circular Dichroism Modeling, a recently developed tool to compute electronic circular dichroism spectra of large macromolecular systems will be presented. Its application to DNA and peptides conformations will be shown and compared to other existing methods.

## Linear absorption

Modeling linear absorption spectra of complex system has been a crucial and very important task and many efforts have been devoted to its realization in the past. In particular the role of the environment should be precisely taken into account when devoting to the study of complex realistic systems.

One of the most straightforward methods to take the environment into accounts has been by means of continuum polarizable methods (Rinaldi and Rivail, 1973; Tomasi, 2004), such as COSMO (Klamt and Schüürmann, 1993) or PCM (Tomasi et al., 2005). Even though the former have been, and still are, extremely popular and proven efficient in modeling homogeneous media, more recently the use of QM/MM methods has gain appeal in particular for the possibility to treat inhomogeneous environment that may include chromophores interacting with complex biological systems, such as nucleic acids and proteins, or materials.

However, going from an implicit to an explicit description of the molecular surroundings, brings important conceptual problems that should be properly addressed, and that are partially different from the one observed for ground state problems. On that respect, indeed, when considering QM/MM methods one can distinguish a hierarchy in the treatment of the environment (Monari et al., 2013; Rivail et al., 2015). In particular one may distinguish mechanical embedding (ME) in which only the geometrical constraints imposed by the MM partition on the QM geometry are taken into account; the electrostatic embedding (EE) in which the polarization of the QM wave function by the MM point charges is allowed; and finally the polarizable embedding (PE) in which the back polarization of the MM potential by the QM partition is included. While, force field parameterization allows to recover the polarization of the bulk when dealing with ground state problems, and hence EE is usually sufficient to achieve a correct description of the complex environment, the situation is much more complex in the case of electronic excited state problems. Indeed, by definition, an electronic excited state will involve a sudden, and in some instance important, change in the electronic density distribution, hence, and as a consequence, the response, i.e., the polarization, of the nearby molecular surroundings will become important and cannot be neglected anymore. One straightforward strategy to include polarizable effects is to switch from a fixed-charge force field to a polarizable one (Caprasecca et al., 2014; Shi et al., 2015). Different strategies exist, based on the inclusion of atom multipole moments in addition to the charges, and have been interfaced with a number of very used codes such as Dalton and Gaussian QM/MMPol (Orlando and Jorgensen, 2010; Loco et al., 2016b). However, the parameterization of polarizable force field can in some instance be rather cumbersome, while the calculation overhead can become important.

**Figure 1:**
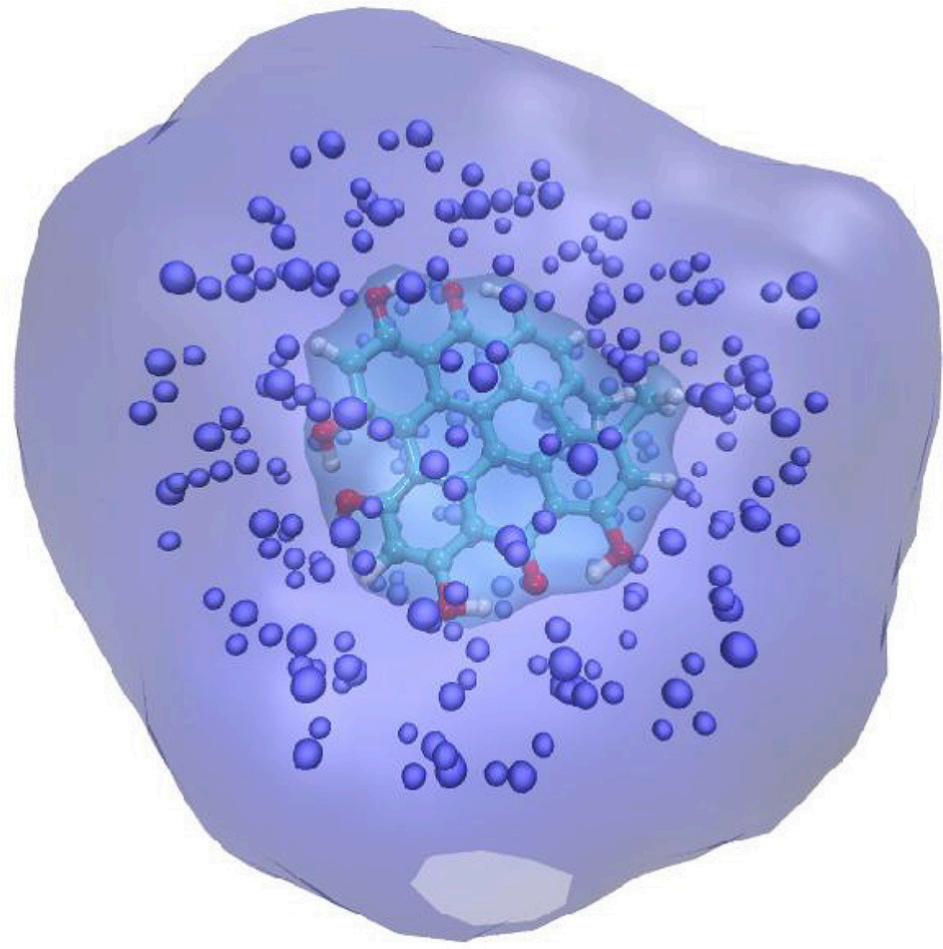
Schematic representation of the QM/MM + ERS partition for a solvated chromophore. The QM chromophore is represented in balls and sticks and atom color, the explicit MM charges are indicates as van der Waals spheres. The QM chromophore is placed in a cavity created in the polarizable continuum that is schematized by the cyan transparent surface.

An alternative strategy developed about 10 years ago (Jacquemin et al., 2009; Monari et al., 2013) and called the electrostatic response of the surrounding (ERS) proposes to tackle polarization issues by surrounding the QM partition in a polarizable cavity described by the fast component of the dielectric constants, i.e., the dielectric constant extrapolated to infinite frequencies. To be precise, let us remind that the former continuum will be embedded in the MM partition but will not interact with the MM point charges (Figure 1). The idea underlining this strategy is due to the fact that the fast component of the dielectric constant represents the instantaneous rearrangement of the surrounding to the change in the QM electronic density, hence the use of a self-consistent reaction field (SCRF) approach, like in PCM, will allow to optimize the surroundings charge distribution to the change induced by the electronic transition. The advantages of this approach are two-fold: first no particular parameterization is required since the fast component of the dielectric constant is almost a constant value comprised between 1.50 and 2.00 a.u. for every non-conductive material. Secondly the computational overhead compared to EE is absolutely negligible and of exactly the same magnitude as a PCM calculation. Those aspects may hence qualify ERS as an efficient and universal strategy to include PE in complex systems. Indeed, its performance has been extensively proven in a number of different systems, including chromophore embedded in protein (Monari et al., 2012a,b), dyes interacting with nucleic acids (Chantzis et al., 2013, 2014; Etienne et al., 2013; Véry et al., 2014; Dumont and Monari, 2015) as well as native proteins (Etienne et al., 2014b), and its capacity to yield extremely accurate results has been confirmed. Even though conceptually generalizable to every quantum chemistry method able to provide electronic excitation energies, most of the applications have been performed in the framework of time dependent density functional theory (TD-DFT) approach. This fact is definitively understandable considering from the one hand the good ratio between accuracy and computational cost, and on the other hand the fact that TD-DFT provides a well balanced description of a relatively large manifold of excited states independently on the choice of an active space. However, as in all DFT methods, the drawback is the necessity to preliminary choose an exchange-correlation functionals whose effects on the accuracy of the results can be rather important. The general good practices concerning the choice of the functionals for excited state calculations that have been established for isolated systems also hold when the environment is taken into account, as an example while hybrid represent an obvious improvement over the performance of pure LDA and GGA functionals, charge-transfer states will necessitate the inclusion of long-range corrected functionals. Furthermore, the use of diagnostic indexes (Peach et al., 2008; Le Bahers et al., 2011; Etienne et al., 2014c) giving a numerical representation of the amount of charge transfer is strongly encouraged.

**Figure 2:**
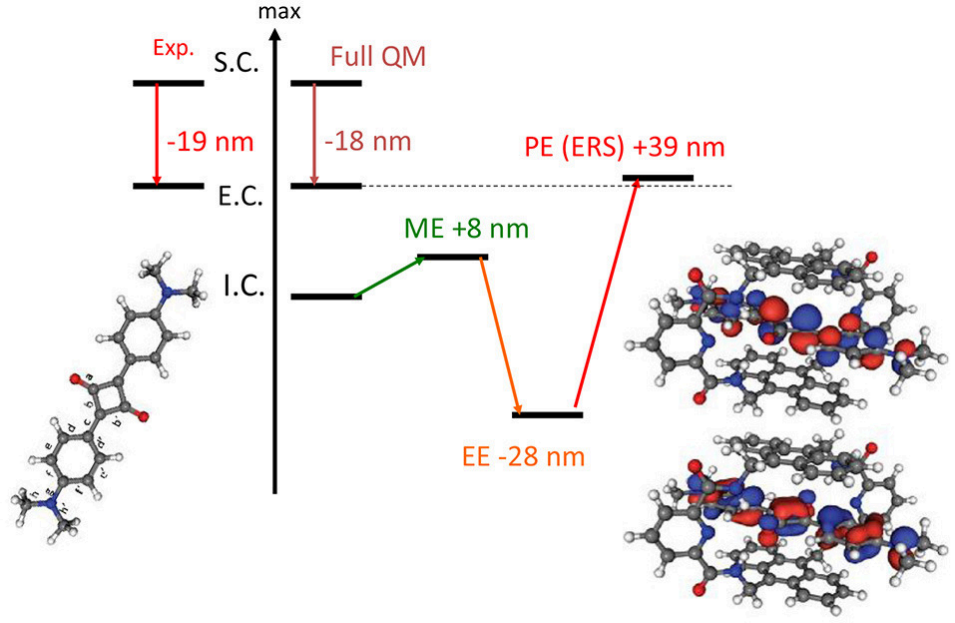
Illustration of the importance of the inclusion of PE in the calculation of absorption spectrum. The results are obtained at TD-DFT level using PBE0 for an encapsulated squaraine as reported by Jacquemin et al. (2009). S.C stands for solvated chromophore, I.C. for isolated chromophore i.e., in gas phase, E.C. for encapsulated chromophore. Full QM refers to the inclusion of both the chromophore and the cage in the QM partition.

Indeed in a most stunning application (Jacquemin et al., 2009), involving the TD-DFT study of a caged dye, the necessity of including PE in the QM/MM calculation of absorption spectra has been absolutely evidenced. It has been shown that the effects of EE and PE where of the same order of magnitude but while the former was inducing a red-shift the latter is blue-shifting the absorption back as compared to the ME results (Figure 2). Hence, the inclusion of EE alone would have strongly deteriorated the quality of the computed results.

Once the necessity to include ME, EE, and PE in the QM/MM spectra calculations has been firmly assessed one has to take into account that, especially in complex systems and complex environments, a good representation of the electronic excited states and hence of the absorption spectra requires to go beyond the usual vertical representation of the excitation energy, in which one consider that absorption or emission spectra can be obtained as vertical transition from the starting state equilibrium geometry. Usually, and as a first approximation, the explicit calculation of the difference in energy between the first vibrational states (E_0−0_) can be used, however this strategy does not allow to take into accounts the full vibrational structure. An alternative strategy is based on the explicit calculation of the quantum based vibronic coupling, i.e., the Franck-Condon and Herzberg-Teller factors, based on the overlap between the vibrational wavefunctions and whose convolution will give the vibronic electronic spectra, i.e., the exact coupling between the vibrational and electronic states (Improta et al., 2007; Santoro et al., 2007). Some codes allowing to calculate such factors exist, and have shown considerable success in particular in the description of the coupling with the high frequency modes and the corresponding change in the spectral band shape (Cerezo et al., 2015, 2016). However, also because of the numerical calculation of the vibrational overlap integrals, they show limitations in the case of low-frequency large-amplitude vibrational modes, such as out-of-plane bending of π-conjugated systems. Furthermore, the explicit inclusion of the quantum effects, provided by the Franck-Condon principle, is less important for the latter mode, which due to the large amplitude behaves much closer to the classical limit. Hence an alternative strategy to take into account such vibrational mode is to perform an accurate sampling of the potential energy surface landscape, allowing for the extraction of snapshots from which vertical excited states can be obtained (Etienne et al., 2013). The final spectrum will then be constituted by the convolution of all the vertical transitions, weighted by the corresponding intensities, for each of the snapshots. Even though the quantum chemistry calculation for the excited states will have to be repeated in order to obtain a reasonable statistical sampling of the conformational space, the explicit calculation of the Franck-Condon factors is avoided. Furthermore, different sampling techniques can be used ranging from classical or QM/MM molecular dynamics, up to semi-classical distributions such as the Wigner one (Dahl and Springborg, 1988). Most notably, in Wigner distribution the snapshots are obtained from the chromophore equilibrium geometry and its vibrational harmonic frequencies, taking into account the energies of a set of quantum harmonic oscillators.

**Figure 3:**
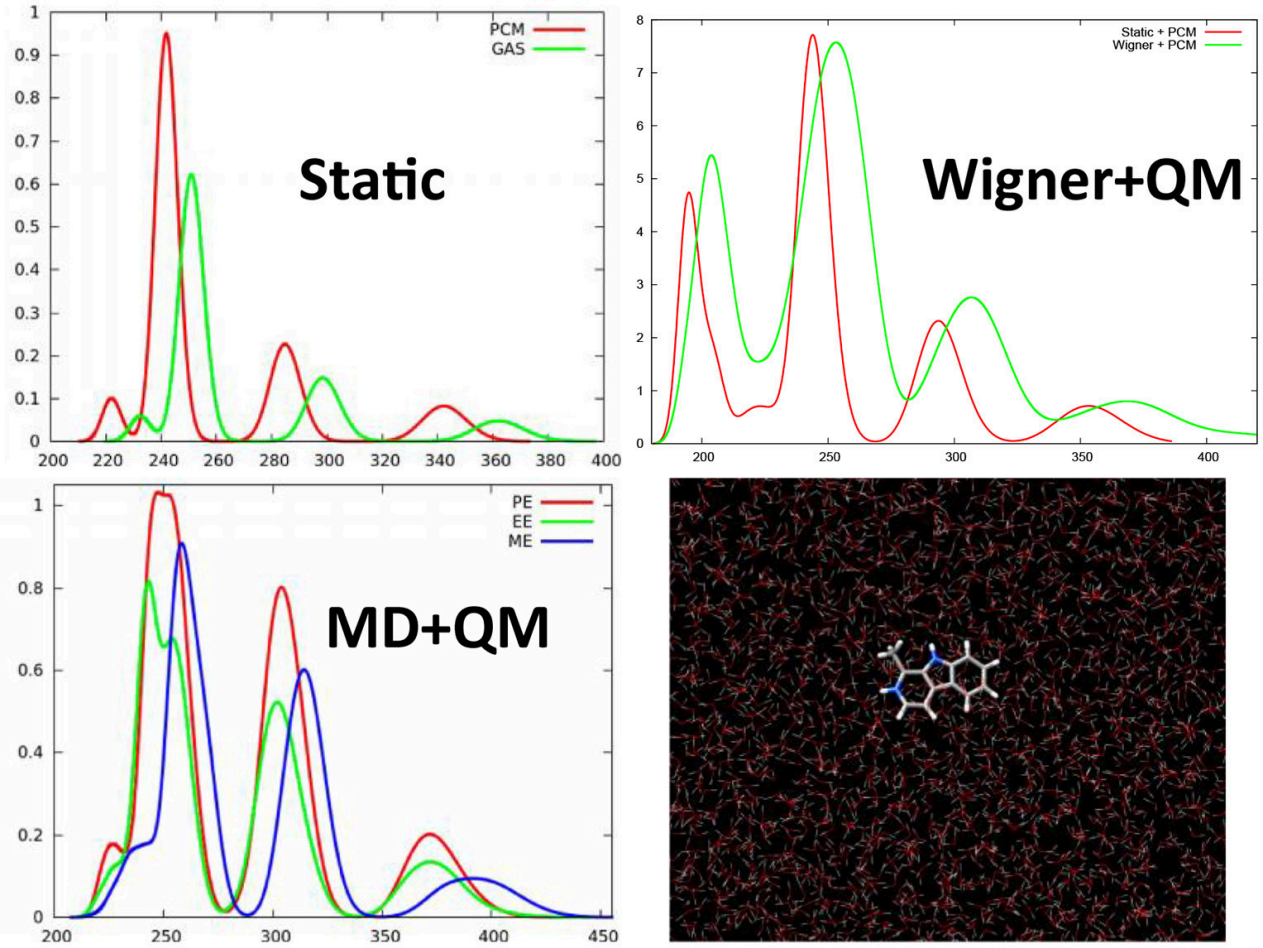
TD-DFT calculated absorption spectra of harmane cation obtained with different strategies to account for environmental and dynamic effects. This includes the static approach, i.e., calculating the spectrum as vertical transitions from the ground state equilibrium geometry, or QM/MM calculation taking into account differentially ME, EE, and PE on top of snapshots extracted from a ground state MD trajectory. Finally the previous results have been compared with the spectrum obtained at QM level with the environment treated at PCM level and the sampling of the ground state conformations performed through a Wigner distribution.

We have shown that even for simple organic molecules such as harmane cations whose spectrum was simulated at TD-DFT level (Etienne et al., 2013, 2014d), the inclusion of vibrational effects via the calculation of the excited states on snapshots extracted from a classical molecular dynamics trajectory in a water box, induces a red-shift of about 20 nm and hence allows to perfectly recover the experimental results (Figure 3). Remarkably enough the same red-shift, and hence the same agreement with experimental results is obtained when the excited states of harmane are computed: (i) at QM/MM level from snapshots extracted from a molecular dynamic trajectory; (ii) at QM + PCM level from snapshots extracted from a molecular dynamic trajectory; and (iii) at QM + PCM level from snapshots extracted from a Wigner distribution. Hence, it appears clearly that the crucial element to recover experimental results will be the correct treatment of vibrational effects rather than the treatment of the environment. However, it has to be underlined that, even though to a less extent than for encapsulated squaraine, EE and PE produce different shifts on the absorption wavelength (Figure 3). The same importance of the dynamic effects has also been found in the calculation of emission, i.e., fluorescence, spectra of harmane (Etienne et al., 2014d). On the same point, and also due to the local nature of the bright states involved in the spectrum the choice of the functional appears as not crucial, and generally hybrid ones may be considered as accurate and provide vertical excitation energies results comparable with the one obtained at equation of motion coupled cluster (EOM-CC) level (Etienne et al., 2014d).

**Figure 4:**
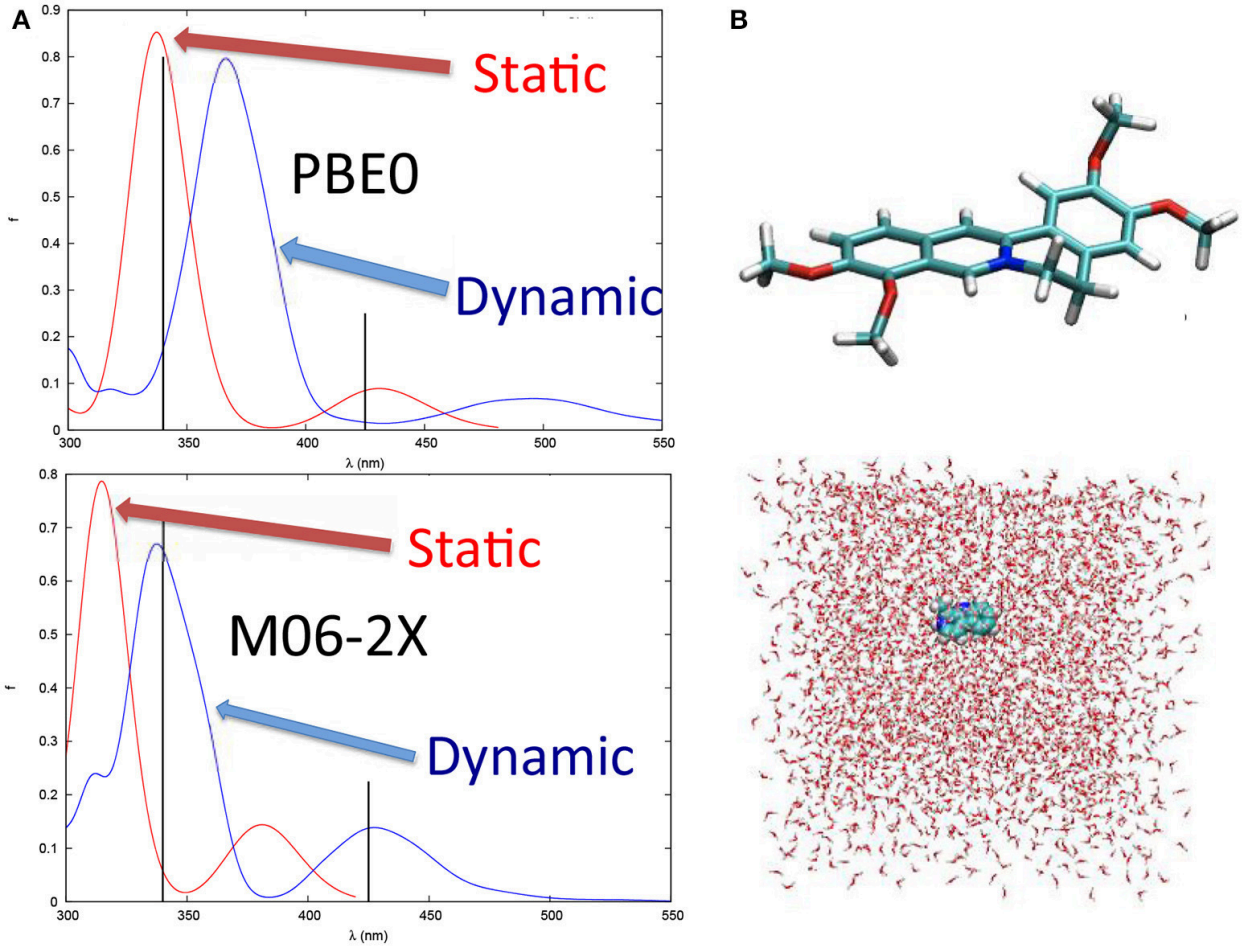
**(A)** The absorption spectrum of palmatine obtained with static approach and at QM/MM level after a MD sampling of the ground state conformational space with PBE0 and M06-2X functionals, respectively. Note that the black sticks represent the experimental absorption maxima. **(B)** Structure of palmatine and of the solvation box used for the MD simulations.

Vibrational effects also play a crucial role in the case of other organic compounds such as the natural occurring drug palmatine (Dumont and Monari, 2015), known to interact with DNA and triggering singlet oxygen production (Hirakawa and Hirano, 2008). It has been shown (Figure 4) that the calculation of vertical transitions from the equilibrium ground state geometry provides an excellent agreement with experimental values when hybrid functionals, such as PBE0, are used. On the other hand, including dynamic effects via a molecular dynamic sampling leads to an excessive red-shift of the spectrum. However, this agreement is only due to the unphysical stabilization of charge transfer states induced by the use of hybrid functional and hence is only related to errors cancelation. Indeed, when using long-range corrected or meta-GGA functionals, the effects are reversed and in the case of M06-2X, the spectrum modeled including the dynamic effects gives results in perfect agreement with the experimental one while the static one appears way too blue-shifted (Figure 4). This tendency can be perfectly rationalized considering that long-range corrected functionals solve the problem related to the overstabilization of the charge transfer states, and hence globally blue-shift the absorption spectrum avoiding error cancelation with the dynamic effects. Hence, palmatine theoretical spectroscopy represent a very nice example of the subtle interplay of different interlocking effects in the treatment of excited states in complex systems, that necessitate to finely tune the computational strategy to avoid spurious errors and a bad description of the dominant states. Furthermore, the sampling of the palmatine conformational space through molecular dynamics has also allowed to recover its interaction modes with DNA and to model the change in absorption spectrum due to this interaction (Dumont and Monari, 2015).

In the same context it has been shown that the inclusion of vibrational and dynamics effects may also be crucial in determining not only the absorption spectrum but also the photophysically allowed pathways of many chromophores. As an example the sampling of the conformational space of the hypericin drug (Gattuso et al., 2017a) via the extraction of snapshots through Wigner and molecular dynamics distribution has allowed to calculate also other crucial parameters such as the spin-orbit coupling (SOC) between the low lying excited singlet and triplet states. In particular it has been shown that the SOC is generally increased due to geometry distribution and may reach values up to some tenths of cm^−1^. This sort of vibrationally allowed SOC can rationalize the relatively efficient intersystem-crossing experienced by hypericin and hence its efficient production of singlet oxygen upon irradiation that constitutes the basis of some of its pharmacological activity. Remarkably enough those properties have been observed both in the case of aqueous solution and when interacting with lipid membrane bilayers.

Hence the combination of a proper treatment of the environment, allowing for the inclusion of environmental and dynamics effects, allows the precise determination of spectroscopic properties as well as photophysical key parameters, of a number of complex systems including optical materials such as solar cells dyes (Sengul et al., 2017) and poly-thiophene units (Turan et al., 2016), as well as biological systems and drugs. Furthermore, the effects of solvent relaxation and their influence on the spectroscopical properties can also be assessed through similar protocols (Zvereva et al., 2018).

## Non-linear spectroscopy

### Two-photon absorption and second-order harmonic generation: general principles, experimental techniques, and computational methods

**Figure 5:**
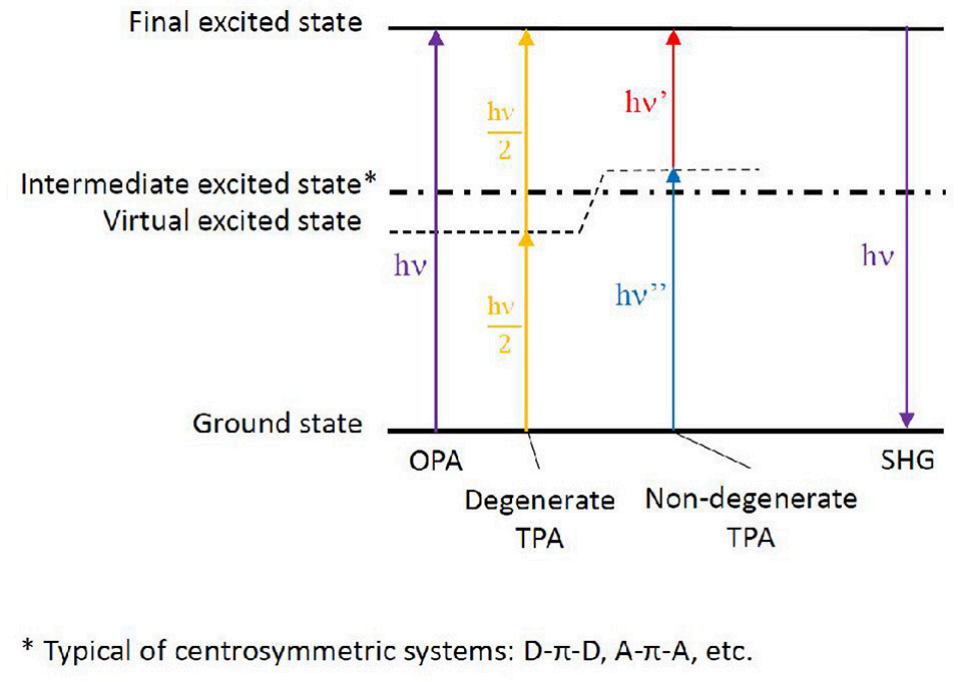
Energy levels scheme depicting one-photon absorption (OPA), degenerate and non-degenerate two-photon absorption (TPA), eventually followed by second harmonic generation (SHG). The virtual state generated by the electromagnetic field of one photon, thus allowing the summation of the second photon, is shown. In the case of centrosymmetric chromophores, an intermediate excited state is present as non-optically bright for TPA. The more this intermediate state is near in energy to the virtual state (hence to the energy of one of the photons), the brighter the TPA strength to the final excited state. The latter phenomenon takes the name of resonance-enhanced TPA.

If linear absorption properties are essential to characterize bio-macromolecules, non-linear absorption processes lead to important features, especially when looking at possible applications. Highly investigated in the past with the goal to enhance materials properties, the scientific interest has recently turned toward bio-macromolecules, in some cases reaching the bio-medicine field. Especially, two phenomena are of utmost interest: multi-photon absorption and high-order harmonic generation. Among them, because of the lower symmetry usually found in biological media compared to condensed materials, two-photon absorption (TPA) and second-order harmonic generation (SHG) are mainly concerned (Antoine and Bonačić-Koutecký, 2018). In both cases, two photons interact simultaneously with the same molecule resulting, in the case of TPA, in the absorption to an electronic excited state corresponding to the sum of the energies of the two incoming photons or, in the case of SHG, in the emission of a photon arising from the sum of the incoming photon energies. Moreover, we can discern between degenerate two-photon phenomena, in which both photons have the same energy (hence same frequency and same wavelength) and non-degenerate phenomena, in which the two photons differ in their energies (Figure 5).

Concerning applications in the biological field, TPA is an especially desired feature since it solves two problems at once:

- The simultaneous absorption of two photons allows, in the case of degenerate TPA, to divide the required energy by a factor of two, i.e., to double the vertical transition wavelength. This permit to notably shift the required incoming photon energy toward the red part of the visible spectrum (i.e., bathochromic shift), eventually allowing to enter in the so-called near-infrared therapeutic window (from 650 to 1350 nm), where the penetration of biological tissues is maximal (Tsai et al., 2001; Mojzisova and Vermot, 2011).
- The probability for the two photons to be absorbed simultaneously is proportional to the square of the light source intensity. This will definitely increase the spatial precision, since TPA will most likely play a role only at the laser focal point, decreasing in intensity much faster than one-photon absorption (OPA), i.e., much faster than linear absorption processes discussed in the previous section. This results in a crucial advantage for bio-medical applications, since TPA can focus precisely on the lesion area, reducing the side effects (Sun and Dalton, 2008; Benninger and Piston, 2013).

On the other hand, SHG is mainly used as a principle to build powerful microscopy techniques of interest in bio-imaging. Hence, the key factor stands in the possibility to generate visible light at high-resolution. This allowed high resolution imaging from deep inside biological tissues, as lipid bilayers of cell membranes, by designing active chromophores with a required balance between hydrophobic and hydrophilic moieties (Barsu et al., 2009; Reeve et al., 2010).

Finally, the use of TPA and/or SHG should also be considered as highly promising in theranostics, a fusion of therapeutics and diagnostics (Jeelani et al., 2014). Indeed, solely TPA coupled to red/infrared emission can constitute the basis of emissive drugs combining treatment and imaging properties. Likewise, specific chromophores, as retinal analogs among others (Theer et al., 2011), can combine TPA and SHG to match absorption, emission, and reactivity purposes (Barsu et al., 2006). In the following, we will focus on non-linear absorption, hence on TPA.

Even though a detailed understanding of the underlying physical principles is out of the scope of the present review, some fundamental concepts should be recalled in order to interpret experimental and theoretical results, thus possibly predict TPA properties. In linear absorption spectra, the intensity values are obtained by the Beer–Lambert law, by which the logarithm of the intensity is proportional to the molar absorption coefficient and to two experimental parameters: the solution concentration and the optical length, i.e., the size of the spectroscopic cell. On the other hand, when theoretically determining linear absorption spectra, the oscillator strength is calculated, a dimensionless quantity proportional to the square of the transition dipole moment between the ground and the excited state of interest. Hence, a straightforward comparison between experimental and theoretical linear spectra is not possible, and usually relies on normalization of the computed spectra. This is not the case for TPA, since absolute Göppert-Mayers units (1 GM = 10^−50^ cm^4^ s photons^−1^ molecule^−1^) can be both measured experimentally and computed by theory, being proportional to the so-called molecular TPA cross-section (Göppert-Mayer, 1931; Kaiser and Garrett, 1961). This makes in principle the comparison between theory and experiment easier, even though much less data are available in the literature compared to OPA, due to the more sophisticated experimental setups and the computational implementations required.

Concerning experiments, after the first demonstration of TPA by organic dyes (Peticolas et al., 1963), different techniques were developed. Indeed, an added requirement makes TPA direct measurements more complicated than OPA ones: the necessity to determine the source irradiance for each probed wavelength. This means that not only the energy of the laser beam, but also its spatial distribution (including possible changes of propagation through the sample) and its pulse-width (including eventual temporal modulation) need to be accurately monitored all over the spectral window to be probed (Negres et al., 2002). The main technique presently in use for TPA direct measurements is the z-scan technique. It derives its name from the required movement of the sample along the z axis defined as the distance between the focused laser and the detector (Sheik-Bahae et al., 1990). Nevertheless, as a way to overcome the difficulties caused by TPA direct measurements, an alternative technique has emerged, based on two-photon excited fluorescence (Xu and Webb, 1996): if OPA and TPA spectra of a reference compound are known (de Reguardati et al., 2016), then a simple comparison of one- and two-photon excited fluorescence spectra between the sample and the reference will allow to cancel out most of the variables required by the direct measurement, finally leading to the TPA spectrum of the sample. Thanks to this indirect approach, it is not necessary to manage all the issues raised by a direct laser focus, even though fluorescence is required in order to detect a signal, hence limiting the amount and type of molecules that can be measured. Moreover, TPA measurements cannot be obtained in the spectral region where OPA is dominant.

As for theory, general rules can be followed to attempt structure-property relationships, even though the inclusion of dynamics effects by molecular dynamics, coupled to explicit solvent and complex bio-chemical environments, can lead to additional and unexplored informations. More in detail, chromophores can be divided in centrosymmetric and non-centrosymmetric ones. Especially, while a π-conjugated backbone is necessary for an organic molecule to absorb light at long wavelengths, donor (D), and acceptor (A) groups can be added to the ends or in the middle of the π-conjugated chromophore, generating centrosymmetric (e.g., D-π-D, A-π-A, D-π-A-π-D, A-π-D-π-A) and non-centrosymmetric (e.g., D-π-A) structures. Indeed, symmetry plays a role in the photophysical transition selection rules (Heflin et al., 1988; Dixit et al., 1991): in centrosymmetric chromophores, a virtual state can be generated while the molecule experiences the field of the first photon, allowing the following—*ca*. 5 fs delay (Birge and Pierce, 1986)—photon to reach the final state, hence favoring TPA over OPA. This is possible thanks to the presence of an intermediate state next to the virtual state, a resonance condition which is generally not fulfilled in non-centrosymmetric chromophores, where in principle TPA and OPA are both possible with the same probability. (Figure 5; Pawlicki et al., 2009). While the presence of charge transfer states can indeed improve TPA cross section, their presence is however not strictly necessary to induce non-linear absorption (Beerepoot et al., 2014).

This can partially explain why, in the past, most of the attention focused on centrosymmetric modified molecules—based initially on *trans*-stilbene (Parthenopoulos and Rentzepis, 1989; Ehrlich et al., 1997; Makarov et al., 2008) and azo-aromatic compounds (Antonov et al., 2003; De Boni et al., 2005) among others. This lead to the development of compounds being more appealing for material scientists compared to biochemists and biologists: natural and bio-inspired systems rarely accomplish the requirement to be totally centrosymmetric; moreover, strong D and/or A groups cannot be always added, depending on the frequently delicate balance between chemical structure and biological function.

Computationally, two-photon transition moments are available for EOM-EE-CCSD (Equation-Of-Motion for Excitation Energies Coupled-Cluster C with Single and Double substitutions; Krylov and Gill, 2013) and ADC (Algebraic Diagrammatic Construction; Knippenberg et al., 2012) methods within the Q-Chem package (Shao et al., 2015), but also at CC2 (second-order approximate Coupled-Cluster singles and doubles model; Christiansen et al., 1995) level of theory through the TURBOMOLE program package (Furche et al., 2014), and at TD-DFT (Time Dependent-Density Functional Theory) through the DALTON2016 program suite (Aidas et al., 2014). In the latter case, since TPA active molecules usually include D and A groups as explained above, it is important to choose functionals which can describe the displacement of charge during the transition from D-centered orbitals to A-centered ones, as hybrid and long-range corrected exchange-correlation functionals.

### Theoretical predictions: level of theory and environmental effects

**Figure 6:**
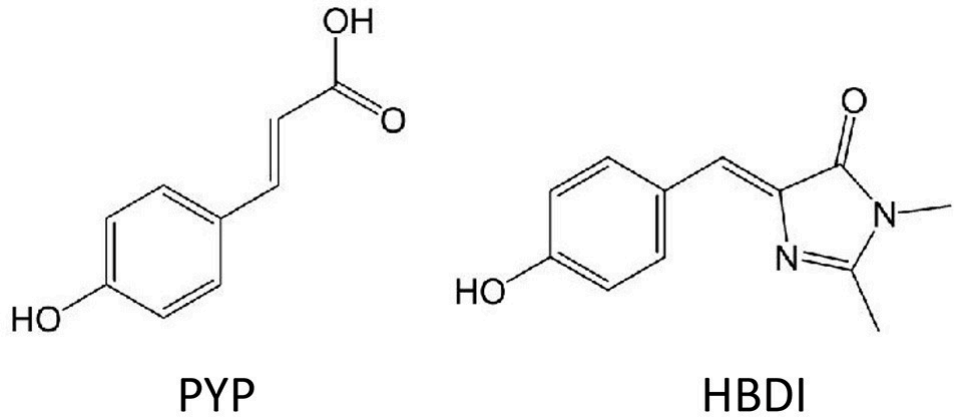
Molecular structures of chromophores which TPA properties were calculated at different theoretical level in gas-phase: HBDI and PYP.

Two benchmark studies compare EOM-EE-CCSD, CC2 and TD-DFT/CAM-B3LYP (Yanai et al., 2004) two-photon cross sections for the chromophores of the Photoactive Yellow Protein (PYP) and of the Green Fluorescent Protein, GFP, in its neutral form (HBDI) (Figure 6; Beerepoot et al., 2015; Nanda and Krylov, 2015). Being all calculations performed in gas-phase, the results point toward a general qualitative agreement between the three levels of theory, with quantitative discrepancies: TD-DFT/CAM-B3LYP TPA cross-sections were found to be 1.5–3 times smaller than EOM-EE-CCSD and CC2 ones (Beerepoot et al., 2015). Of course, the divergence could lie in the different description of the excited state dipole moments (Bednarska et al., 2013). Nevertheless, also because the effect of the environment is not taken into account by this study, conclusions cannot be raised on the basis of a comparison with experimental results.

On the other hand, the computationally more affordable TD-DFT (compared to Equation-Of-Motion and Coupled Cluster approximations) allows to treat the environment by PCM and QM/MM methods at a much easier cost, including dynamics effects, finally allowing to establish how the TPA maximum intensity is affected by a realistic environment. Moreover, the spectral window and spectral shape can be recovered, determining the applicability of the chromophore for therapeutic purposes or as constituent of bio-materials.

**Figure 7:**
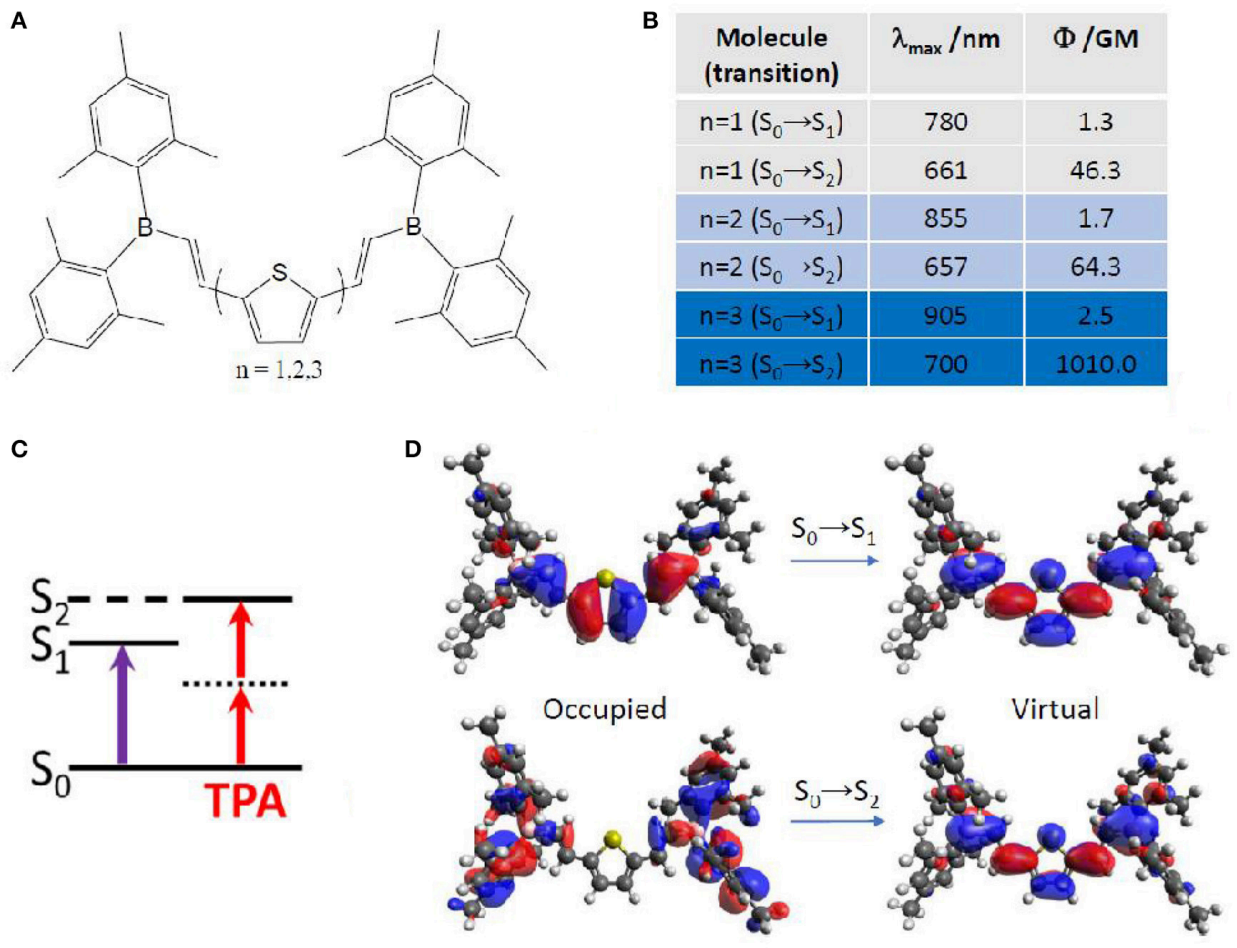
**(A)** Boron containing arenes under study, including mono-, di- and tri-thiophene in the core of the chromophore. **(B)** Corresponding TPA energies and strength for the lowest two excited singlet states, S_1_ and S_2_. **(C)** Simple energy diagram showing that OPA is preferred to S_1_, while TPA is preferred to S_2_, with S_1_ functioning as an intermediate state, as expected for (almost) centrosymmetric molecules. **(D)** Natural transition orbitals for the chromophore containing mono-thiophene.

More in detail, TPA properties of boron containing arenes were evaluated at TD-DFT level, including the solvent by linear response PCM (Turan et al., 2016). In this case, a vertical approach was used, meaning that all molecules were optimized on the ground state followed by calculation of the vertical transition to the excited state. The results point toward a S_2_ optically bright state for TPA, while S_1_ is the bright state for OPA (Figure 7). This can be explained in terms of molecular orbitals. Especially, electronic density reorganization in the excited state can be efficiently monitored through Natural Transition Orbitals (NTOs) (Martin, 2003), in this case obtained with the Nancy_EX code (Etienne et al., 2014a,c). The S_0_→S_1_ transition is of local π,π^*^ character, while the S_0_→S_2_ transition clearly shows the charge transfer from the *E*-dimesitylborylethenyl lateral groups toward the central part of the conjugated chromophore, resembling to a A-π-A centrosymmetric system mentioned in the previous section. Indeed, S_0_→S_1_ TPA values are almost negligible (not more than 2.5 GM), while S_0_→S_2_ TPA values reach up to 1010 GM. Interestingly, we should mention that TPA values for this kind of compounds is predicted to change by two orders of magnitude, when looking at the type and number of arenes contained. Especially, thiophene based compounds correspond to the highest cross section values, compared to phenyl and fluorinated phenyl. Moreover, increasing the number of linearly linked thiophene rings induced a strong non-linear effect, passing from 46 GM (one thiophene) to 64 GM (two thiophenes) to 1010 GM (three thiophenes). In this last case, the experimental value is available [1500 GM (Entwistle et al., 2009)], denoting an underestimation by the TD-DFT/PCM prediction, but keeping a good qualitative agreement.

**Figure 8:**
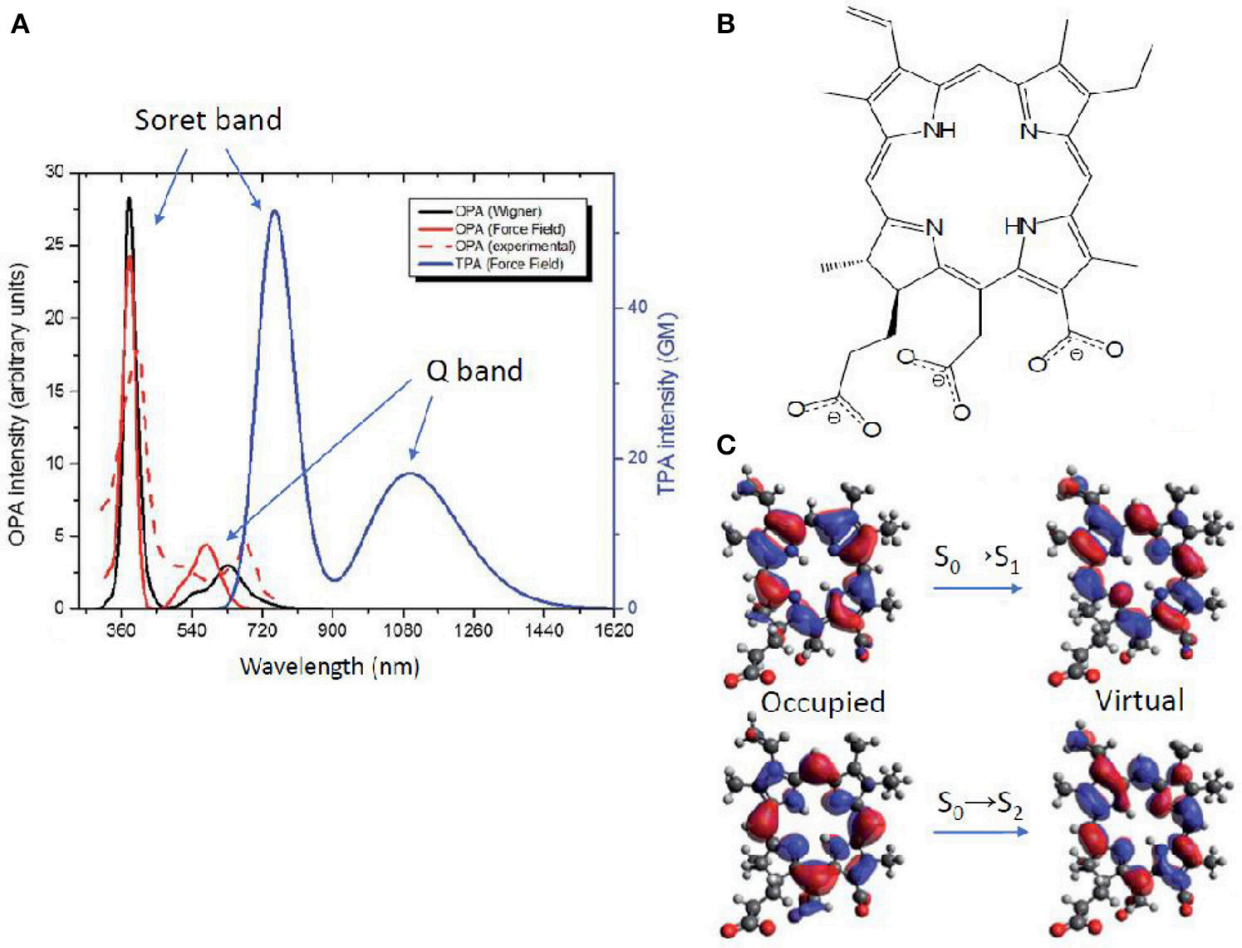
**(A)** Chlorin-e6 absorption spectra: OPA resulting from Wigner distribution and convolution over 20 structures, including water as solvent by PCM (solid red); experimental OPA (Paul et al., 2013) in water as a normalized spectrum (dashed red); OPA (black) and TPA (blue) calculated by QM/MM, convoluting over 20 MD snapshots **(B)** Molecular structure of chlorin-e6 in its anionic form. **(C)** Natural transition orbitals describing transitions to S_1_ (Q band) and to S_2_ (Soret band).

An alternative method to obtain TPA properties is by performing classical MD, followed by QM/MM TPA calculations of single snapshots, randomly selected along the MD trajectory. This approach, explained also in section Linear Absorption for OPA, has the advantage to include the solvent explicitly, even though usually force field parameters have to be extracted for the chromophore under study. Especially, the proper parameterization of chromophores with an extended cyclic π-conjugated backbone is far from being trivial (Li et al., 1989; Autenrieth et al., 2004; Zhang et al., 2012), as is the case of porphyrin-like systems. Indeed, the correct description of low-frequency modes is crucial, since they are expected to notably impact the prediction of the TPA spectrum. An example is chlorin-e6, already reported as photodynamic and antibacterial drug (Zenkevich et al., 1996; Fernandez et al., 1997; Nyman and Hynninen, 2004; Paul et al., 2013; Winkler et al., 2016). In this case, a careful validation of the force field was performed by comparison with the Wigner distribution approach, when calculating OPA properties (Gattuso et al., 2017b). Specifically, a maximization of the overlap between force field and Wigner computed absorption spectra, coupled to a minimization of the Root Mean Square Deviation (RMSD) was accomplished. This force field optimization further allowed TPA properties calculations in explicit water at QM(TD-DFT)/MM level. The spectrum (Figure 8) shows two peaks corresponding—as for all OPA spectra of porphyrin-like systems—to the Soret band (at *ca*. 730 nm and 60 GM) and to the Q band (at *ca*. 1100 nm and 20 GM). TPA intensity values are therefore much lower than boron containing thiophenes aforementioned (Turan et al., 2016), even though still acceptable for bio-applications. Lower TPA intensities can be expected since chlorin-e6 partially loses the centrosymmetry of porphyrins, and can be further rationalized based on its NTOs: both OPA and TPA are possible, with a Soret (S_0_→S_2_) band much more intense than the Q band (S_0_→S_1_) in both cases. Indeed, the NTOs describe for both electronic transitions a charge reorganization centered in the core ring, also explaining the lower change in TPA intensity compared to the three orders of magnitude observed for thiophene based chromophores (Figure 7). Anyway, only a relatively small overlap between OPA and TPA spectra is expected, thus justifying the use of the indirect—and easier to afford—experimental setup, also thanks to the fluorescent properties typical of these molecules (Gattuso et al., 2017b; Liu et al., 2018). This could hence encourage more experimental groups to measure this type of molecules, to better assess theoretical methods and proposed advancements in various applications (Ryu et al., 2018).

**Figure 9:**
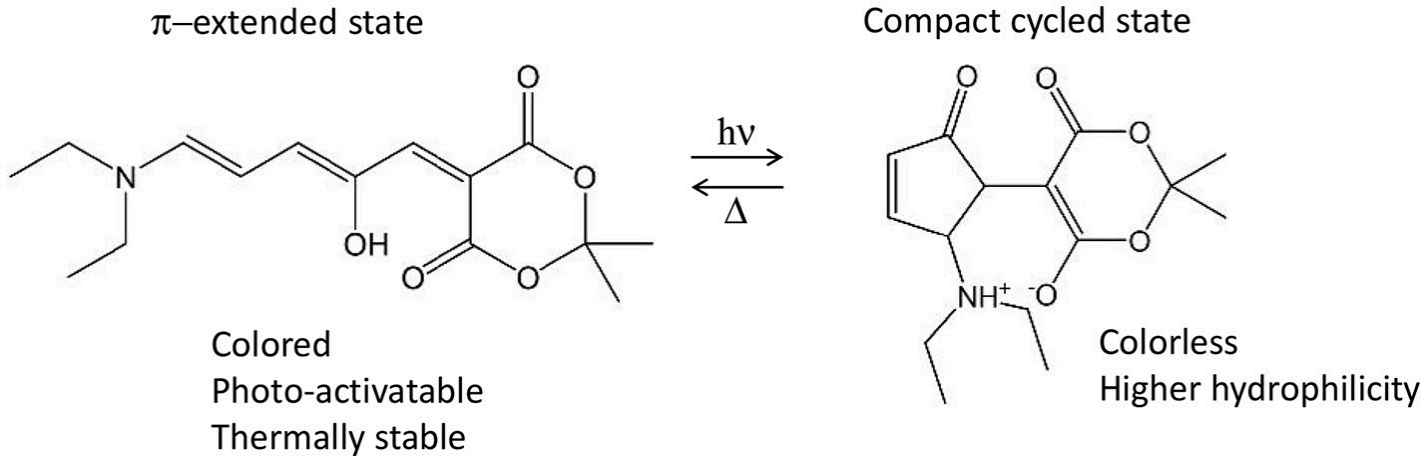
Example of Donor-Acceptor Stenhouse Adduct (DASA), including its reactivity and main properties associated to both states. In this example, the non-centrosymmetric D-π-A molecule includes a diethyl amine moiety as donor and the Meldrum's acid as acceptor.

Nonetheless, several photo-active chromophores are totally non-centrosymmetric. Examples are the retinal and the Donor-Acceptor Stenhouse Adduct (DASA). The former is known as the *cis*-*trans* photo-isomerizable switch of rhodopsins, G-protein coupled receptors responsible for the process of vision (Wald et al., 1968; Okada et al., 2004) and recently employed as optogenetic tools (Deisseroth, 2011; Tischer and Weiner, 2014; Guo et al., 2016; Hontani et al., 2017). The latter is a recently discovered type of photo-switch (Helmy et al., 2014a,b) which converts from an initial π-extended colored state into a final compact colorless state, moreover accompanied by a notable polarity change (Figure 9). Both retinals and DASAs are characterized by a lowest-lying singlet excited state (S_1_) of partial charge transfer character, even though of different origin: retinal is a protonated Schiff-base which, in biological media, is surrounded by the opsin pocket of rhodopsin, i.e., an active site where the charges and polarity of the amino acid side chains enhances the S_1_ charge transfer character (a sort of induced D-π-A system), compared to a retinal molecule in solution. In the case of DASAs, no macromolecular entity is responsible for the efficiency of its photo-activity, since it is a D-π-A system itself. This symmetry reasonings explain why for both systems a low S_0_→S_1_ TPA intensity is theoretically predicted [*ca*. 2 and 3.32 GM for 11-*cis* retinal and DASA, respectively (Palczewska et al., 2014; García-Iriepa and Marazzi, 2017)]. Even so, retinal isomerization by TPA is expected to play a role to trigger human infrared vision (Artal et al., 2017). Hence, some additional QM/MM studies including polarizable embedding could be worth. Also, it should be mentioned that bio-mimetic photo-switches based on retinal are available, even though still limited to the blue/UV part of spectrum (Sampedro et al., 2004; García-Iriepa et al., 2013, 2016), and could hence greatly benefit from computational design to improve TPA properties. In this context it is also important to precise that cross-sections of <10 GM can indeed be detected experimentally, however their increase is fundamental in order to permit the use of TPA switches in practical applications.

**Figure 10:**
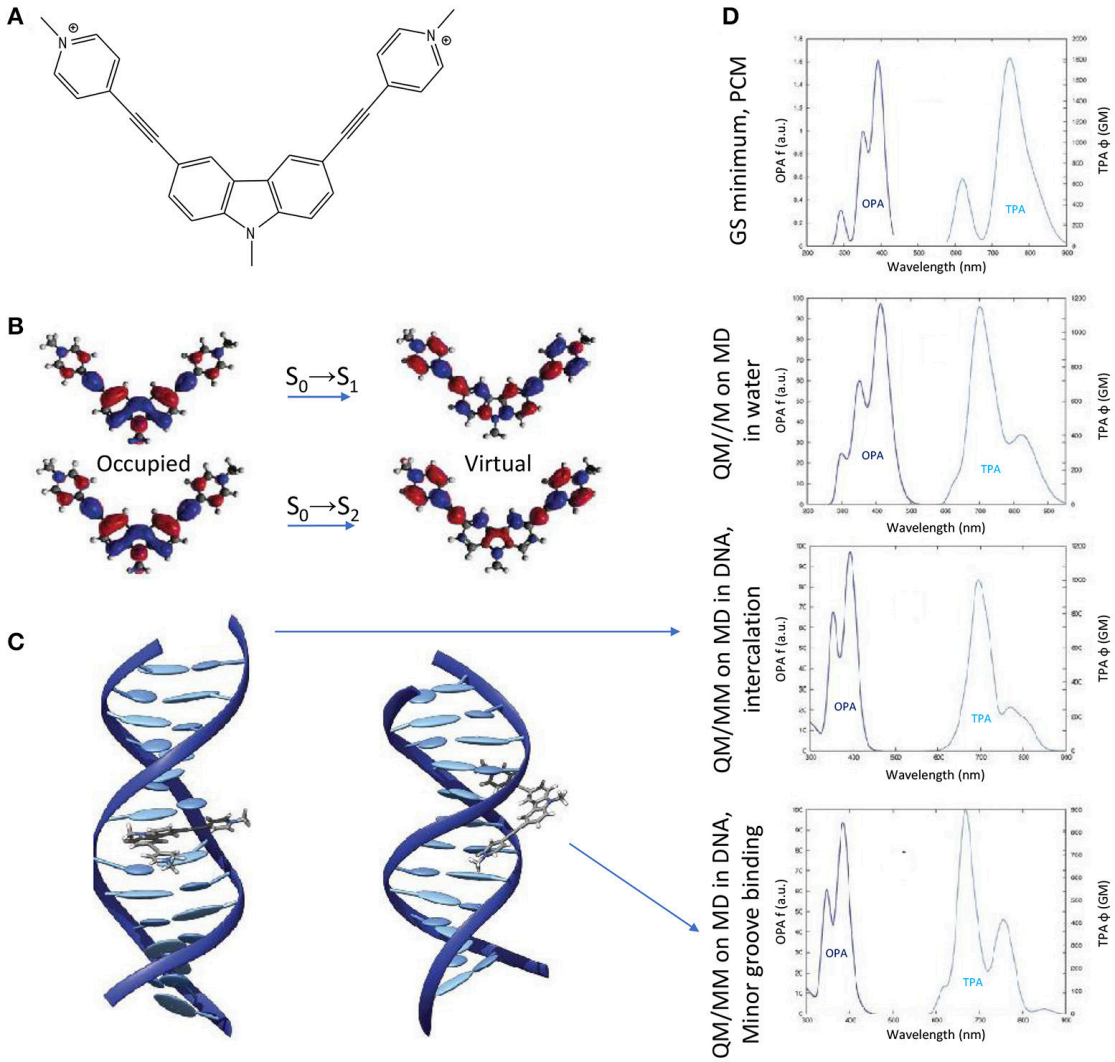
**(A)** Molecular structure of BMEMC. **(B)** Natural transition orbitals for the two lowest-lying optical transitions. **(C)** Main BMEMC-DNA intercalation (left) and minor groove binding (right) modes. **(D)** OPA and TPA spectra corresponding (from up to down) to: the static approach including the solvent by the PCM; a dynamic study including water explicitly; the main DNA intercalation mode; the main DNA minor groove binding mode.

Finally, the impact of the biological media—in the specific case of B-DNA—on the TPA properties of an organic photosensitizer, was established by QM(TD-DFT)/MM calculations (including electrostatic and mechanical embedding), after having determined the photosensitizer-DNA interaction modes by classical MD. Especially, a centrosymmetric A-π-D-π-A dication was selected as photosensitizer (the 3,6-bis[2-(1-methylpyridinium)-]9-methylcarbazole, abbreviated as BMEMC) as it was experimentally shown to cause DNA strand breaks upon infrared irradiation, also in hypoxic conditions (Zheng et al., 2015). The BMEMC-DNA interaction modes and the sensitization mode of action (Gattuso et al., 2016a; Figure 10) were then rationalized on theoretical basis: BMEMC can undergo a photoinduced spontaneous ionization, leading to the production of a solvated electron and a radical cation. Since it is a centrosymmetric structure with relatively strong A groups, TPA intensity values are high, overcoming 1000 GM for the S_0_→S_2_ transition, when computed in water. We should note that in this specific case a proper spectroscopic description of TPA properties is possible only by averaging QM/MM results over a reasonable number of MD trajectory snapshots (20 in this case). Indeed, when compared with the static approach, i.e., by considering only the Franck–Condon structure obtained with the PCM model, we note an apparent inversion of S_0_→S_1_ and S_0_→S_2_ bands in the TPA calculated spectrum (Figure 10). This can be explained in terms of low-frequency modes that are strongly affecting the TPA active S_2_ state, for which only a dynamical approach results in a proper description. Indeed, as shown by the NTOs, the S_2_ virtual orbital is the main responsible for the electron displacement toward the pyrimidinium A edges, which are also the most affected by low-frequency modes. When looking at the interactions with DNA, four stable binding modes are found by MD: two intercalation modes and two minor groove binding modes, depending if the carbazole D core or a pyrimidinium A edge attempts first the contact with the bio-macromolecule. The QM/MM TPA spectra calculated in the four cases, by convoluting the same number of trajectory snapshots as in water, shows that the non-linear response of BMEMC does not significantly changes in presence of DNA, regarding both spectral regions and peak intensities. Such description matches qualitatively the experimental measurements (Zheng et al., 2015). Comparing intercalation modes, the only notable difference regards the S_0_→S_1_ band shape, broader and less structured in intercalation than in minor groove binding. Again, this is the result of electrostatic and mechanical embedding: in intercalation, the core of BMEMC is in the hydrophobic pocket in the middle of DNA, and it is more constrained by the environment. Even if the present example is showing that the impact of the biological media is affecting TPA cross-section only marginal and is by the way more oriented toward photodynamic therapy than diagnosis, it is important to remind that possible applications of TPA fluorescence, and its modulation by the environment, are of great importance for bio-imaging and are paving the way to a new dimension in theranostic, i.e., the combined treatment and diagnosis (Hu et al., 2018).

### Effect of the polarizable embedding scheme

The polarizable embedding scheme has become recently available to include not only polarization of the QM region due to MM charges, but also vice-versa, thus reaching an even more realistic description of the environment. Especially, TD-DFT can be applied to calculate multi-photon absorption properties in a modified version of the DALTON program (Steindal et al., 2016). This can definitely improve the quality in the treatment of the environmental effects, even though increasing the computational cost. Indeed, the same neutral GFP chromophore previously studied in gas-phase (Figure 6; Beerepoot et al., 2015) was found to absorb with a bathochromic shift (of 0.09–0.16 eV) when including the polarizable embedding scheme for two-, three- and four-photon absorption. Nevertheless, we should note that TPA strengths are more sensitive to the size of the QM region than OPA strengths (Steindal et al., 2016), hence envisaging two alternatives to maintain a reasonable balance between computational quality and cost: (i) increase notably the size of the QM region, keeping an electrostatic QM/MM embedding scheme; (ii) keep an acceptable size of the QM region, applying a polarizable QM/MM embedding scheme. Moreover, for future tests and benchmarks, alternative QM/MM border treatments rather than the hydrogen link-atom approach could be proposed, as a way to limit the size of the QM region and improve the quality of TPA properties prediction.

## Circular dichroism modeling

Even though linear and non-linear absorption spectroscopies proved to be efficient in probing the binding propensity of organic compounds with complex bio-macromolecular systems (Jiang et al., 2014; Li et al., 2015), they are unable to provide information about the global structure of bio-macromolecular systems. Indeed, the sensitivity of these techniques is not sufficient to differentiate between different arrangements. In contrary, electronic Circular Dichroism (CD), which is a fast and sensitive spectroscopic method, provides specific optical signature for each polypeptide secondary structure arrangement (alpha-helix, beta-sheet, random coils,) (Greenfield, 2007) or nucleic acids macromolecular structure (A/B/Z-DNA, G-quadruplexes, i-motifs, and more; Kypr et al., 2009; Vorlíčková et al., 2012). Hence, it allows an unequivocal differentiation of the systems configurations. Moreover, since the resulting optical signal is directly correlated to the global structure of the system, specific phenomena can be efficiently probed such as the formation of aggregates between molecular compounds and proteins or DNA. It can be used to investigate structural reorganizations from a configuration to another induced by external effects. Moreover, in the case of DNA/ligand and DNA/protein binding, CD is able to provide structural information at two levels: (i) on one side, considering the spectral fingerprint of well-characterized macromolecular arrangements, the structural modifications (local or global) induced by the binding can be probed by simply following the spectral features while increasing the concentration of the substrate (Basu and Suresh Kumar, 2014); (ii) on the other side, the induced CD (for non-chiral compounds) or band shape modifications (for chiral compounds) while bound to a biomolecular system can also provide insights in the binding configurations or be used to perform spectroscopic titration and hence recover binding free-energies (Holmgaard List et al., 2017).

In the case of nucleic acids aggregates, (Kypr et al., 2009; Vorlíčková et al., 2012) extensive experimental studies of the ability of CD to compare and differentiate most of the well-known conformations such as A/B/Z-DNA, guanine, and cytosine quadruplexes or even i-motifs have been reported. Moreover, the results demonstrated the efficiency of CD to follow induced transitions between two configurations. Indeed, they described the trifluoroethanol-induced transitions from B-DNA to A-DNA (sequence poly[GCGGCGACTGGTGAGTACGC]) and B-DNA to Z-DNA (sequence poly[d(GC)]). Also, they described the evident spectral modifications during either transitions between quadruplexes or the formations of i-motifs while varying the pH or increasing the ionic strength. This work demonstrated how robust the information given by CD can be and paved the way to more advanced studies on DNA/ligand and DNA/protein bindings.

Considering natural nucleic and amino acid arrangements such as B-DNA or alpha helices, their CD spectral features arises from the interaction of optically active chromophores organized in a specific layout. The chromophoric units, giving rise to the most prominent CD signals, are the nucleobases in the case of DNA, and the -(N-H)-(C = O)- peptide bonds in the case of proteins. Assembled in larger macromolecular ensembles owning intrinsic chirality (such as the B-DNA double helix or alpha helical peptides) the interaction between these achiral molecular chromophores will induce circularly polarized optical signals. The interactions influencing the electronic properties of these units can either be (i) hydrogen bonds, (ii) π-stacking interactions, (iii) electrostatic interactions. Furthermore, upon the optical excitation of the aggregate one can evidence, (iv) excitons formation or (v) population of Charge-Transfer (CT) states. In the case of B-DNA, the delocalization of excitons is not extended to more than three stacked nucleobases and doublets of stacked nucleobase pairs can be considered as a model accurate enough to provide a reasonable description of the B-DNA CD spectra (Nogueira et al., 2017) as will be presented in the following sections.

Modeling the CD spectra of large macromolecular systems such as proteins and DNA is challenging since it requires to overcome numerous technical drawbacks, some of them being common also to the calculations of linear and non-linear absorption but being emphasized by the inherent extreme sensitivity of CD spectroscopy to subtle structural and electronic effects:

- Sufficient and accurate sampling of the conformational space. The increase of computing resources allows nowadays extensively long Molecular Dynamics (MD) simulations, reaching from microsecond to millisecond time scales, directly comparable to experimental results (Galindo-Murillo et al., 2015). Moreover, the accuracy of current force fields describing these biological systems have been demonstrated and the sampled potential energy surfaces can be considered close to *in-vitro* experiments (Dans et al., 2017). The recently available parmbsc1 correction (Ivani et al., 2015) available for AMBER is currently one of the most robust force field for nucleic acids.
- Computation of the excited states. TD-DFT, even considering its limitations, is a widely employed computational tool for obtaining of electronic properties in biological systems. It has proved to be accurate enough to be comparable with experiments (Laurent and Jacquemin, 2013). Also, more advanced methods such as CASSCF, CASPT2, or CC have been employed but still suffer from their much higher computational cost which is a drawback when considering large biological systems made up of hundreds to thousands of atoms.
- Taking into account the environment. Even though the use of common continuum models such as PCM or COSMO, provide relatively accurate results and are computationally cheap, many specific cases have required to consider explicit solvations to account for more specific interactions between solvents and the molecular region of interest. This is overcome using hybrid QM/MM methods.

The protocols that have been employed by our group to model CD spectra of biomolecular systems will be described more in details in the following. Nevertheless, they can be summarized in four steps: (i) the starting structure can be obtained from X-ray or NMR studies or from *in-silico* builders of biomolecules (NAB, tleap Case et al., 2017); (ii) MD simulation to sample the potential energy surface of the system; (iii) computation of excited states at *ab-initio* level and eventually of their coupling over the full macromolecular system via semiempirical Hamiltonians; (iv) convolution of excited states energies and transition strengths using Gaussian or Lorentzian shaped functions. In particular concerning the crucial point of the coupling between the individual chromophores we chose to use the simplest dipole moment approach. Hence, the coupling is estimated simply by the scalar product of the transition dipole moments calculated for each of the individual chromophores weighted by the distance of their center of charges. This strategy differs from the one employed by other authors (Jurinovich et al., 2014) who chose to explicitly determine the coupling via the excited states' density matrices, and although much simpler both conceptually and from a computational point of view is able to yield quite accurate representation of the macromolecular CD signal. However, care should be taken in using the approximate dipole model, especially in case of strongly coupled systems, for instance closely packed chromophore, for which the explicit calculation of the coupling via the density matrix approach may be necessary to avoid the model breakdown. Indeed, it is known that for distances of the interacting units smaller than 5 Å an overestimation of the excitonic coupling by the dipole model can be experienced.

### Nucleic acids

#### Circular dichroism of B-DNA

The specific CD signals of B-DNA are directly associated to its famous double helical structure. Modeling the CD spectral features that can be directly matched with experiments is still far from being straightforward and suffers many drawbacks: (i) most experimental studies of phenomena in DNA are performed on non-specific sequences such as calf thymus DNA (Kankia et al., 2001). Since the CD signal is directly related to the sequence, it is thus impossible to provide a meaningful model without considering all the possible nucleobases sequences. Moreover, in the case of the interaction of DNA with an organic molecule, the binding will often be non-specific. Since the length of B-DNA strands that are commonly modeled using MD ranges from 10 to 32 base-pairs, the number of possible binding sites increases drastically, thus reproducing every possible binding configuration becomes an unfeasible task. Based on this, model systems must be considered made up of a selected sequence with a specific binding configuration (Basu and Suresh Kumar, 2014). That is why, most of the CD modeling that have been performed so far were of either well-characterized sequences (such as the X-ray crystal structure—pdb code: 1BNA–or NMR structure—pdb code: 1K9H) or of the simplest nucleic acid sequences, being hetero- and homo-polymers of adenine (A)-thymine (T) and guanine (G)-cytosine (C) (Drew et al., 1981).

The first reported modeling of B-DNA CD employing *ab-initio* methods has been performed by (Miyahara et al., 2013) on the structure of a poly(dGCCCGGGC) double strand obtained by X-ray crystallography (Heinemann et al., 1987). In this study, the excited states energies were computed using the Symmetry-Adapted Cluster (SAC)-Configuration Interaction (CI) theory after pre-optimization of the system at the DFT/B3LYP/6-31G(d,p) level. Solvation has been taken into account using Polarizable Continuum Model (PCM) implemented in Gaussian. In this work, either eight excited states have been computed in dimer models (two stacked nucleobases to study the influence of π-stacking interactions or a nucleobase pair including hydrogen bonding) or 14 excited states have been considered in a tetramer model made up of two stacked nucleobase pairs (containing both π-stacking and hydrogen bonding interactions). For the smaller dimer models, it has been demonstrated, on one side, that hydrogen bonding within base pairs is accountable for the change in excitation energies compared to single nucleobases and, on the other side, that π-stacking is responsible for the sign of the CD signal. Finally, the computation performed on the tetramer model resulted in a CD spectrum in very good agreement with experiment.

Employing a Complex Polarization Propagator (CPP), Di Meo et al. modeled the CD spectra of a poly(dA.dT)_20_ B-DNA double strand (Di Meo et al., 2015). Excited states of model base pairs dimers and trimers have been computed at the TD-DFT/CAM-B3LYP/aug-cc-pVDZ level of theory considering 30 excited states. Also, the sampling of the B-DNA conformational space has been performed through 100 ns long MD simulations using the amberff12-bsc0 force field. Their results show a very good agreement with the experimental CD fingerprint of the adenine-thymine base pairs homo-polymers in the near-ultraviolet region, corresponding to two positive bands at 260 and 283 nm and a more intense negative band at 249 nm. Furthermore, Norman et al. (2015) tackled the case of the 147 base pairs long nucleosomal DNA (pdb code: 1KX5) employing a similar procedure. The results demonstrated the influence of the super-helical configuration on the CD spectra, including a hypsochromic shift of the band at 269 nm and a strong decrease of its intensity.

Another approach by Padula et al. to model the CD spectrum of two 10 base-pairs long B-DNA guanine-cytosine hetero- and homo-polymers, relied on the explicit calculation of an exciton coupling in the DNA double strand (Padula et al., 2016). Experimental structures obtained by X-ray crystallography or NMR have been used as starting configurations. The excited states were computed at the TD-DFT/M06-2X/6-31G(d) level of theory taking into account the environment effects and interactions between chromophores using the MMPol method, which allow to treat the environment through a polarizable embedding. Moreover, only bright π-π^*^ excited states have been selected in this protocol. Afterwards, the excitonic Hamiltonian was generated coupling the full transition densities of each partition to obtain, after its diagonalization, the final CD spectra. The final spectrum results (averaged over 270 snapshots extracted from a 90 ns long MD simulation employing the parmbsc0 force field for DNA) recover the overall band shapes and is comparable with experiment.

Our protocol for the simulation of nucleic acids' CD signals, also relied on the 4 steps described hereinbefore with first 10 ns of MD simulation on an *in-silico* built 15 base pairs long DNA double strand (bsc0 force field for nucleic acids). Considering that excited states in B-DNA are relatively local, they can be treated in the framework of the Frenkel excitons theory. In such case, excited states can be computed on a series of decoupled partitions (for example single nucleobases) which will be recoupled by building an exciton coupling matrix. Each excited state for each partition will be coupled with all the other excited states using the methodology presented in (Gattuso et al., 2015, 2016b). The excited states have been obtained using TD-DFT/M06-2X/6-311+G(d) on 40 snapshots, divided in up to 8 different partitions made up of nucleobase pairs.

**Figure 11:**
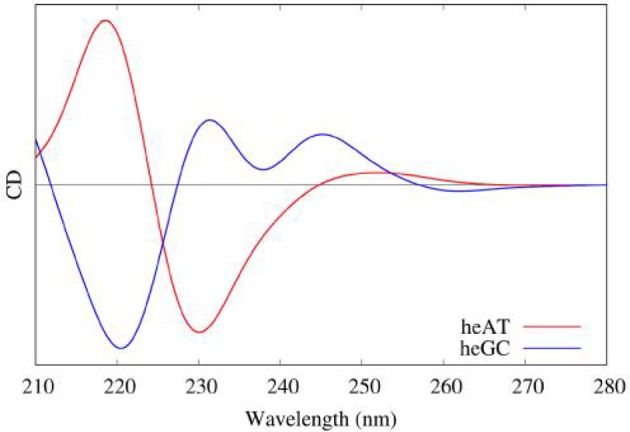
Modeled CD spectra of a hetero-polymer of adenine-thymine (red) and a heteropolymer of guanine-cytosine (blue) base pairs.

Moreover, QM/MM embedding was used to take into account the environment, in order to tune the electronic properties of each partition. Polarization was accounted using ERS as presented before. Then the resulting rotatory strength have been convoluted using Gaussian shaped functions of 0.2 eV Full Width at Half Maximum (FWHM). The results obtained on hetero-polymers of AT and GC base pairs are presented in Figure 11 and nicely underline the sequence effects on the DNA CD spectra.

#### Circular dichroism of G-quadruplexes

G-quadruplexes (G4) can be sorted in three families,—parallel, hybrid, and antiparallel—corresponding to their specific nucleic acids arrangements with each family possessing sub-members (Randazzo et al., 2012). Their CD spectra have been deeply described (del Villar-Guerra et al., 2017) and can be easily decomposed in a series of nucleic acids interactions components with a major contribution from the central guanine core. Each family can be differentiated by a specific sequence of spectral bands: (i) parallel, characterized by a positive band at 264 nm and a negative one at 245 nm; (ii) hybrid, characterized by positive bands at 295 and 260 nm and a negative one at 245 nm; (iii) anti-parallel, characterized by a positive band at 295 nm and a negative one at 260 nm.

In (Loco et al., 2016a) a similar procedure as in (Padula et al., 2016), has been used to model the CD spectrum of an antiparallel G4 (pdb code: 143D). However, this study did not consider a sampling of the conformations using MD simulations, but was instead performed directly on the experimental (crystallographic) structure. In the excitonic Hamiltonian, guanines have been considered as individual partitions brought in interaction using the MMPol polarizable embedding (TD-DFT/M06-2X/6-311+G(d) level, 10 excited states calculated for each guanine). The resulting spectrum almost matches the experiment one.

**Figure 12:**
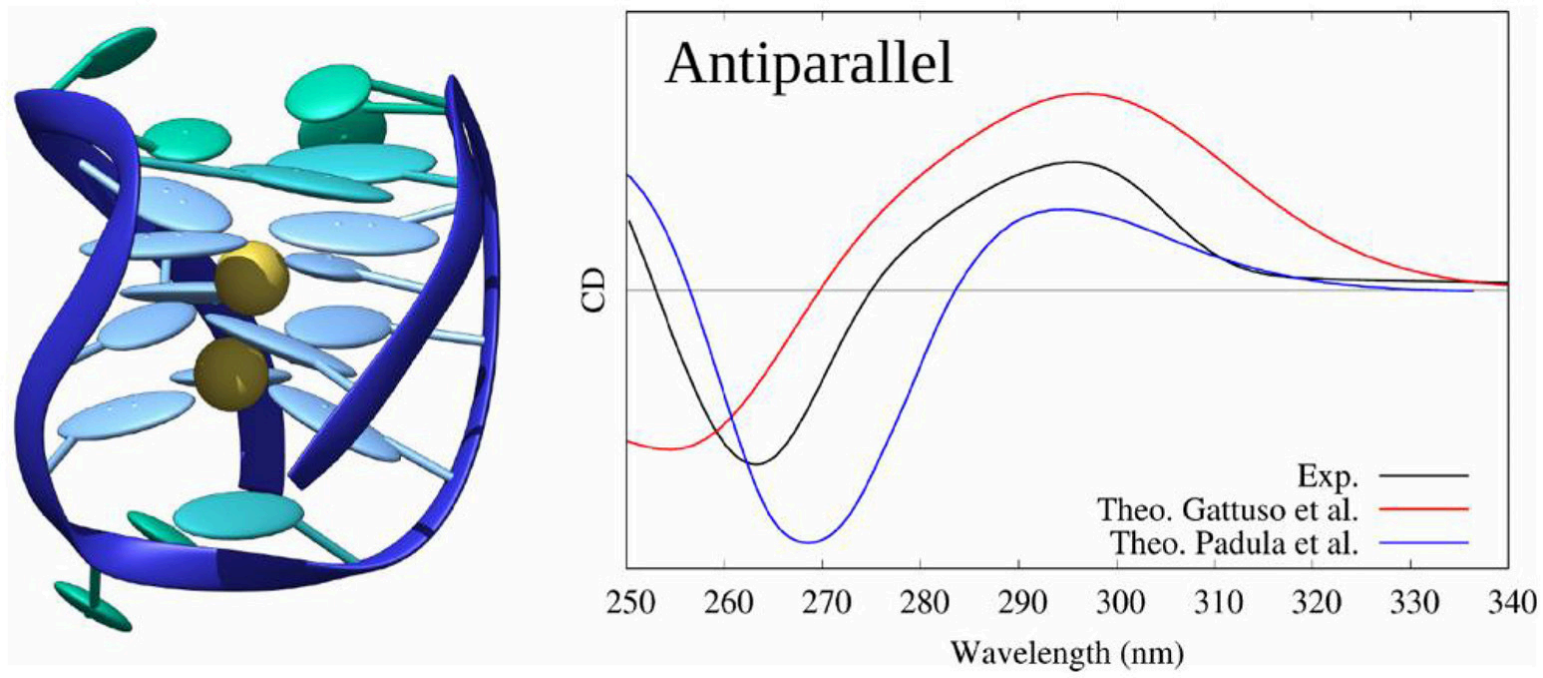
Experimental and modeled CD spectra of the antiparallel G4 structure (pdb code: 143D).

Using the same protocol as for B-DNA, including sampling the conformational space through MD, and considering as partitions the four triplets of stacked guanines of the G4 core, we also proved (Gattuso et al., 2016b) that the excitons coupling gives a satisfactory simulated CD spectrum of the three G4 families with a clear evidence of their spectral features (see Figure 12). Our starting structures were X-ray crystal structures (pdb code: 1KF1 for parallel, 2HY9 for hybrid and 143D for antiparallel configurations) which possess the main spectral features detailed before. Moreover, our results demonstrated that to model the CD spectra of G4, the explicit environment has to be considered with great care, since these aggregates are stabilized by two central cations (K^+^ for hybrid and parallel configurations and Na^+^ for the antiparallel one) electrostatically bound to the guanines of the core.

### Peptides

#### Circular dichroism of alpha-helices

Circular dichroism of protein secondary structures also draws strong interest since it can be, as DNA, directly related to the global structure of the bio-system (Greenfield, 2007). Indeed, it provides information about open and closed conformations in the case of binding with a ligand, or even protein-protein and protein-DNA bindings (Carpenter et al., 2009). Moreover, CD can probe reorganizations in the case of environmental changes, such as increase of ionic strength or temperature (Jirgensons, 1980). So far, the main attempts to model the CD spectra of macromolecular amino acids arrangements have targeted alpha helices and their specific spectral band shapes, consisting of two negative bands at 208 and 222 nm and an intense negative band at 193 nm.

Kaminský et al. (2011) performed the first CD modeling of relatively long alpha helices. Their protocol previewed to first sampling the conformational space of *in-silico* built alpha helices using MD simulations, followed by a computation of the excited states and rotatory strengths. Especially, Ac-(Ala)_*n*_-NH-Me structures (with *n* going from 2 to 19) were studied by Transition Dipole Coupling (TDC) and TD-DFT, using the B3LYP functional and various basis sets, focusing on the difference between vacuum and the COSMO method to include solvent effects. Even though the resulting band shape is reasonably comparable with experimental CD, the MD simulations has been performed on *in-silico* built starting configurations for a maximum of 50 ps. Considering the high flexibility and necessity to allow the system (and solvent) to reorganize, this time length cannot definitely be considered as sufficient to sample the ensemble of configurations of such system. They mainly concluded that considering several consecutive amino acids is important to allow exciton delocalization.

**Figure 13:**
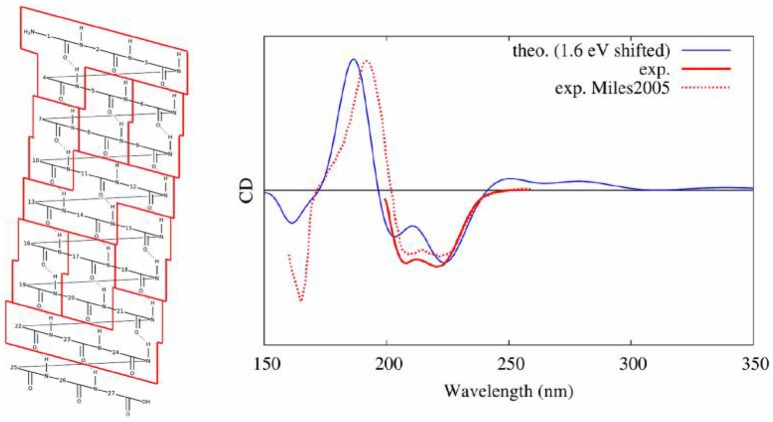
Experimental (Miles and Wallace, 2006) and modeled (Gattuso et al., 2017b) CD spectra of an alpha helical peptide. On the left, the selected partitioning scheme to apply the Frenkel excitons scheme is shown.

Our protocol (Gattuso et al., 2017b), described hereinbefore, combined, as it was the case for nucleic acids, conformational sampling through MD, followed by the diagonalization of an excitonic coupling Hamiltonian whose elements are obtained by QM/MM calculations on individual subunits. In detail, in the case of a polypeptide comprising 27 amino acids and assuming a dominant alpha-helix conformation, we performed: (i) 80 ns of MD sampling using amber99 force field; (ii) computation of excited states properties on partitions at the TD-DFT/M06-2X/6-311++G(d,p) level of theory (each computation was performed using hybrid QM/MM method including ERS); (iii) coupling of excitons transition dipole moments; (iv) convolution of rotatory strengths by Gaussian shaped functions of 0.4 eV at FWHM. In this study, we demonstrated that the final CD spectra are highly sensitive to the partitioning scheme with a convergence obtained for loop subunits (four consecutive amino acids, see Figure 13). Also, the simplicity of the Hamiltonian requires to consider with great care the arrangement of partitions. Indeed, the most accurate results were obtained considering a hybrid partitioning including loops of amino acids (quadruplets) and hydrogen bounded dimers (couples). In fact, this allowed to retain the higher accuracy obtained for bigger molecular partitions, while keeping an alpha-helical arrangement of subunits' centers of charges. Most notably, the coupling of MD sampling and QM/MM calculations, also allowed to recover the modification of CD signals induced by the partial breaking of the alpha-helix, due to the coupling of the polypeptide with a photoactive switch undergoing *cis*/*trans* isomerization.

## General conclusion

In this review we gather recent work done in our group dealing with the description of electronic excited states in complex molecular systems. We present three main applications, namely linear absorption, non-linear optical properties (mainly two-photon absorption) and circular dichroism together with the specific methodological development that has been made. We show that when an adequate description of both surrounding and dynamical effects is chosen, our calculations agree nicely with the available experimental data and can thus be used for prediction when experimental data are missing, or at least, as in the case of the structurally sensitive electronic CD, they allow for a simple and straightforward one-to-one mapping with the system structural features. As a general take home message, one should be aware that a good sampling of the conformational space and a polarizable embedding scheme are required to obtain reliable results.

## Author contributions

AM: was responsible for the part on linear absorption; MM: took care of the non-linear optical properties; HG: handled the section devoted to circular dichroism; XA: was responsible for the remaining parts of the manuscript.

### Conflict of interest statement

The authors declare that the research was conducted in the absence of any commercial or financial relationships that could be construed as a potential conflict of interest. The reviewer, AN, declared a past co-authorship with several of the authors, XA, AM, MM, to the handling Editor.

## Footnotes

^1^Available online at: https://www.nobelprize.org/nobel_prizes/chemistry/laureates/2013/ (Accessed Jan 27, 2018)
